# Composition and Biological Activity of Colored Rice—A Comprehensive Review

**DOI:** 10.3390/foods14081394

**Published:** 2025-04-17

**Authors:** Mingchao Zhao, Xiaorong Xiao, Dingsha Jin, Linan Zhai, Yapeng Li, Qingwen Yang, Funeng Xing, Weihua Qiao, Xiaowei Yan, Qingjie Tang

**Affiliations:** 1 Institute of Food Crops, Hainan Academy of Agricultural Sciences/Hainan Key Laboratory of Crop Genetics and Breeding, Haikou 571100, China; zhaomingchao@webmail.hzau.edu.cn (M.Z.);; 2 Sanya Institute, Hainan Academy of Agricultural Sciences, Sanya 572025, China; 3 National Nanfan Research Institute (Sanya), Chinese Academy of Agricultural Sciences, Sanya 572024, China

**Keywords:** anthocyanins, black rice, brown rice, phenolics, flavonoids, pigmented rice, red rice, rice metabolomics

## Abstract

Colored rice (black, purple, red and brown) has been consumed in China for nearly 4000 years. Recent research has focused on exploring its nutritional and metabolomic profiles and associated health benefits. Due to the improvement in detection and quantification techniques for health-promoting compounds and their activities, the number of studies has increased significantly. In this regard, a timely and updated review of research on nutritional composition, phytochemistry, and metabolite content and composition can significantly enhance consumer awareness. Here, we present a detailed and up-to-date understanding and comparison of the nutritional and phytochemical (metabolite) composition of colored rice. While earlier literature reviews focus on either single type of colored rice or briefly present nutritional comparison or bioactivities, here we present more detailed nutrient profile comparison (carbohydrates, fats, proteins, amino acids, minerals, and vitamins), together with the most recent comparative data on phytochemicals/metabolites (flavonoids, anthocyanins, fatty acids, amino acids and derivatives, phenolic acids, organic acids, alkaloids, and others). We discuss how metabolomics has broadened the scope of research by providing an increasing number of detected compounds. Moreover, directions on the improvement in colored rice nutritional quality through breeding are also presented. Finally, we present the health-beneficial activities (antioxidant, anti-inflammatory, antimicrobial, hypoglycemic, neuroprotective, anti-aging, and antitumor activities) of different colored rice varieties, together with examples of the clinical trials, and discuss which bioactive substances are correlated with such activities.

## 1. Introduction

Colored rice is divided into three categories. The first category is colored rice, that is, rice grains with color (red, brown, green, violet, black, or purple), which is due to the accumulation of anthocyanins in the pericarp and seed coat [1]. The second category refers to the leaves of rice that are red, purple, yellow, or mainly red and purple. Some varieties of this type of colored leaf also have colored grains, but most of the rice is ordinary white rice, mainly used for landscapes and marking. The third category has a colored husk, which means that the leaves of rice are the same as ordinary white rice, but the husk is brown or red. This category, when shelled and polished, gives colored and white kernels [2]. In this review, “colored rice” will refer to those having a colored seed or husk (purple-black, brown, and red). Colored rice is cultivated in China [3], India [3], Indonesia [4], Japan [5], Korea [6], Philippines, Thailand, United States of America, Brazil [7], and some other countries [6]. Historically, black rice has been cultivated in China for more than 4000 years. Black rice was considered “Imperial rice” by the ancient Chinese and was mostly consumed by the emperors for their better well-being [7]. This ancient belief has gained recent attention and several studies have reported that colored rice shows higher antioxidative and free radical scavenging activities compared to white rice [8,9]. The proven health benefits of colored rice have shown their utility in a broad range of industries, i.e., food, pharmaceuticals, and health [10,11,12]. Generally, colored rice is used for wine making, vinegar, porridge, snacks, dumplings, landscape, rice cake, bread, rice noodles, dessert, cookies, rice rolls, rice milk, and rice vegetable salad. Moreover, colored rice is also used as functional food [4,7]. Indigenous knowledge in the geographies where colored rice is grown is being supplemented with scientific evidence to elucidate the roles of the functional molecules present in these types of rice [13]. A timely literature survey of such reports requires a continued effort to keep the consumers informed on the recent scientific developments. While there are several reports/reviews available on the anthocyanin biosynthesis in colored rice [14], nutrient profiles [15], range of bioactive compounds, and their activities [16,17], none has combined and discussed its detailed nutrition, phytochemistry, metabolites, and bioactivities. In this review, we first describe the characteristics of each type of pigmented rice supplemented with information on the anthocyanin biosynthesis pathway. Next, we comparatively describe a detailed and up-to-date nutritional profile of colored rice together with white rice. We describe in detail the individual nutrients, along with their content, including carbohydrates, proteins, fat, lipid, minerals, and vitamins. To do so, we searched relevant literature from Google Scholar, Web of Science, and PubMed by using relevant terms, i.e., pigmented rice, colored rice, comparison between pigmented rice, nutritional profile, and nutrient comparison. Only the articles that showed the most relevant information, such as nutritional profile, were included. Preferably, we surveyed those articles which provide a comparative analysis between white and colored rice types. Notably, recent developments in rice metabolomics [18] have revealed a range of biologically important compounds that impart health benefits [19]. This can open up new fundamental and applied research avenues on personalized nutrition and functional food development [18]. To develop such recommendations for food and medicine, it is important to compare and understand the range of data in the published literature. However, a limited or non-detailed literature survey has been conducted on the compound classes detected through metabolomics-assisted techniques. Considering this, we have reviewed the most recent literature on compound classes, i.e., flavonoids, lipids, amino acids and derivatives, phenolic acids, organic acids, and alkaloids, other compound classes, and volatile aroma compounds. We present the comparative data on their content/composition in different colored rice varieties and highlight the key factors that affect their content and composition. Finally, we present and discuss the latest comparative results on the biological activities of the colored rice. Moreover, we highlight the key limitations in current research and outlay future directions.

The colors of the rice grain, hull, and bran (red, brown, purple, and black) are mainly due to the accumulation of anthocyanins and proanthocyanidins [14]. The accumulation of anthocyanins in rice organs is a domestication-associated trait and researchers often use it for evolutionary analyses. Anthocyanin biosynthesis in rice, like other plants, occurs as a part of the flavonoid biosynthesis pathway. For more details, see a detailed review on how rice organs are colored [14]. The dominant anthocyanin in pigmented rice is cyanidin-3-O-glucoside. Other anthocyanins found in rice include cyanidin-3-gentiobioside, cyanidin-3-rhamnoglucoside, cyanidin-3,5-diglucoside, and cyanidin-3-rutinoside [20,21]. Other than these pigmented rice varieties, golden rice also obtains its color because of pigment biosynthesis, i.e., the carotenoids of carotene and xanthophyll [22]. However, our review does not focus on golden rice and readers are referred to [22,23] on how the golden color is formed and the health benefits associated with it.

**Figure 1:**
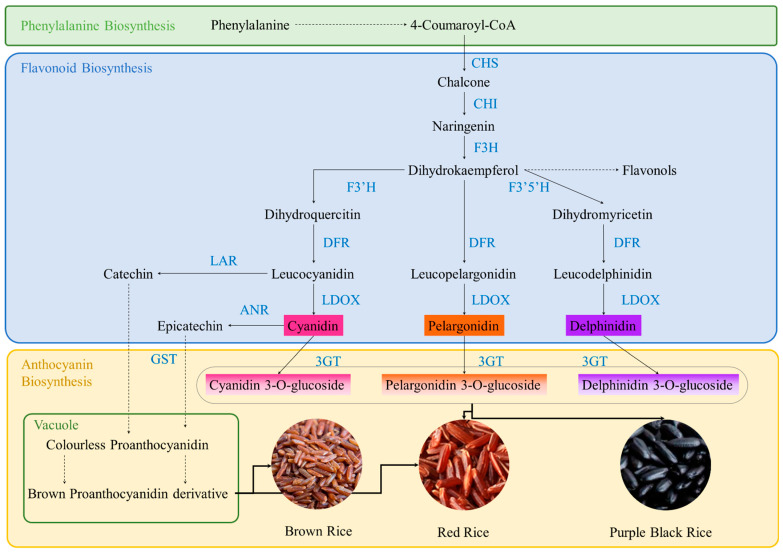
Anthocyanin biosynthesis pathway in colored rice that leads to development of color in rice. CHS, chalcone synthesis; CHI, chalcone isomerase; F3H, flavanone 3-hydroxylase; F3′H, flavonoid 3′-hydroxylase; F3′5′H, flavonoid 3′5′ hydroxylase; DFR, dihydroflavonol 4-reductase; LDOX, leucoanthocyanidin oxidase; 3GT, 3-glucosyl transferase; LAR, leucoanthocyanidin reductase; ANR, anthocyanidin reductase; GST, glutathione S-transferase. The pathway was drawn according to the KEGG pathway (https://www.genome.jp/pathway/osa00942; accessed on 16 October 2024) and a previous report [14].

Black rice is a whole grain rice. Naturally, its seed coat, pericarp, and endosperm (in the case of genetically engineered varieties) are purple-black [24]. The grain is deep black in color and after cooking turns dark purple, and is hence also called purple rice. Black rice grains are found both in *Oryza indica* and *Oryza japonica* species. Black rice is also divided into glutinous and non-glutinous categories. The taste is somewhat nutty. Sensory evaluation of cooked black rice has shown attributes such as smokey, popcorn, sweet aromatic, grain/starchy, bittern, sewer, floral, astringent, sour, sweet, corn, green beans, and dairy [25]. The dark purple-to-black color develops due to the accumulation of anthocyanin-3-O-glucosides, which are biosynthesized via reduction of dihydroflavonols to leucoanthocyanidins and finally to anthocyanin-3-O-glucosides. These steps are carried out by dihydroflavonol reductase (DFR), leucoanthocyanidin oxidase (LDOX), and 3-glucosyl transferase (3GT), respectively (Figure 1). Major anthocyanins reported from black rice are cyanidin-3-glucoside, peonidin-3-glucoside [26], paeoniflorin-β-glucoside, cyanidin-β- and 5-diglucoside, and cyanidin-β-rutoside [27]. Of these, cyanidin-3-glucoside has the highest content (88% to 100%) [20,21]. The color range of the purple-black rice depends on the anthocyanin content in the seeds. Different research groups have reported that black rice has a wide range of total anthocyanin content, i.e., 27.2 to 5045.6 µg/g [1,28]. In addition to anthocyanins, carotenoids and chlorophyll have also been reported in black rice. The major carotenoid in Korean black rice was found to be all-E-lutein (7.14 µg/g). Other carotenoids such as zeaxanthin have also been detected in black rice from Korea [29], Japan [30], and France [31]. However, anthocyanins are still the major pigments responsible for the purple-black color.

Brown rice is also a whole grain rice with the hull removed, while the bran and germ layers remain on the grain. The color of the bran and germ layers are brown; therefore, it is called brown rice. After the milling or whitening process, it becomes white rice. The brown hull color is produced as a result of biosynthesis of proanthocyanins. Proanthocyanin synthesis starts with the reduction of leucoanthocyanidin or cyanidin via catalysis by leucoanthocyanidin reductase (LAR) or anthocyanidin reductase (ANR), respectively, and the products are transported to the vacuole to produce brown proanthocyanin derivatives such as hesperetin 5-O-glucoside, rutin, and delphinidin 3-O-rutinoside (Figure 1) [32]. Usually, the anthocyanin content in brown rice is lower than in other types of pigment rice.

Red rice has a red pericarp. Red rice is available as full or partially hulled varieties. Its flavor is nutty. The pigments responsible for red color formation in this pigmented rice are anthocyanins. Several reports have also documented the role of proanthocyanins in color formation in red rice. The major anthocyanins in red rice (hull or bran or whole rice) are cyanidin-3-O-glucoside, malvidin, peonidin, cyanidin 3-O-(6-O-p-coumaryl) glucoside, peonidin-3-glucoside, and cyanidin di-glucoside [33]. Research on red rice has indicated proanthocyanin content was up to 3060.6 µg/g. In addition to the seed and hull, anthocyanins are also accumulated in rice bran. Red-brown rice also contains small amounts of carotenoids such as lutein, zeaxanthin, β-carotene, and lycopene [34]; for example, Korean brown-red rice contains a very low amount of lutein 0.62 µg/g in [35].

## 2. Nutritional Composition of Colored Rice

### 2.1. Carbohydrates, Protein, and Fat

Nutritional components in colored rice, like white rice, are carbohydrates, proteins, fats, minerals, amino acids, and vitamins. Among these, carbohydrates are the major nutritional components, accounting for ~80%, followed by proteins, fats, and fiber [36]. Brown and red rice have a higher carbohydrate content compared to purple-black rice, and hence provide higher energy values of 367 and 408.6 kcal per 100 g, respectively (Table 1). The main carbohydrates in rice are amylose, with a low molecular weight, and high-molecular-weight amylopectin. Their content in rice determines several characteristics such as relative crystallinity, swelling power, gelatinization temperature, starch digestibility, hardness, taste, and digestibility [37]. A comparison of colored, white, and waxy rice varieties has shown that black and brown rice have higher amylose contents, i.e., 21.8% and 20.5%, respectively [38]. However, it is not an universal observation, as brown indica rice has 9% lower amylose content than white indica rice, signifying that cultivar identity (genetic background) is an important factor [39]. A large-scale nutritional profiling study of 60 Chinese paddy rice varieties, including purple, red, and black varieties, showed that purple rice was richer in sucrose than the others, whereas, red rice was richer in fructose content, followed by black, white, and purple rice [40]. Black rice varieties with different amylose contents, e.g., waxy, medium-amylose (16.03%), and high-amylose (27.14%) contents have been developed, offering different water mobility and texture in preboiled rice [41]. Nevertheless, varieties differing in their amylose and amylopectin content differ in their glycemic index. For example, a study using brown, red, black, and white rice reported the highest amylose content (30.03%) in brown rice, while the three other types had non-significant differences. However, white rice exhibited the highest glycemic index (69.74), followed by red, black, and brown rice [42]. Therefore, the starch profile can affect the glycemic index of pigmented and non-pigmented rice [43,44]. Although researchers focus on reducing the glycemic index in white rice [45], pigmented rice could be a useful nutritional alternative. Diets with a low glycemic index are considered effective at glycemic control by reducing cholesterol, fasting glucose, body mass index, and low-density lipoprotein [46]. Although rice breeders are currently focusing on breeding low-glycemic-index rice cultivars [47], the pigmented rice varieties with a low glycemic index are potentially superior than white rice in controlling postprandial glycemia. Studies on purple [48] and red rice [49] have indicated their ability to prevent activities in experimental diabetes models and human subjects or in vitro. Thus, broadening the usage of pigmented rice, in addition to low-glycemic-index white rice [50], offers a healthier diet alternative to counter the diabetes epidemic. Breeding efforts on pigmented rice with a further-reduced glycemic index should be a priority in this regard. Most researchers are focusing on breeding low-glycemic-index white rice, and have identified that amylose content is significantly negatively correlated with the predicted glycemic index. Moreover, several glycemic-index-associated genes, genomic regions, and markers have also been identified [51,52,53]. The knowledge accumulated in white rice breeding can be expanded to further lower the amylose content in pigmented rice.

Protein is the second major component of rice with exceptional digestibility and nutritional profile. A balanced protein and carbohydrate content is beneficial to consumers’ health. Within colored rice, variation in protein content exists such that purple-black rice contains the highest protein content (9.4 g) per 100 g, followed by red and brown rice. The digestibility of rice protein is mainly due to the higher proportion of lysine [15]. Whole purple rice contains ~20% higher total soluble protein content compared to red, golden, and white rice [54]. This higher protein and amino acid content in colored rice is because the essential amino acids are also present in rice bran, and its inclusion in the diet can ensure higher protein and amino acid contents compared to white rice, which is mostly used polished [55,56]. However, differences between varieties and cultivars also result in variable protein content owing to nitrogen fertilizer use, genotype, environment, or abiotic stress impact [57]. Major rice proteins are glutelin, prolamin, globulin, and albumin [3]. A study of the composition and content of black rice (100 g) proteins showed that glutelin (7.929 g) was the main protein component followed by albumin (1.208 g), globulin (0.327 g), and prolamin (0.119 g). In the case of brown rice, the relative contributions of fractional proteins, i.e., globulin and glutelin, are higher than those of albumin and prolamin in Indian brown and white rice in terms of fraction and percentage [58]. Thus, glutelins are the most abundant fraction of proteins in all types of colored or white rice. They have superior nutritional value than others because of higher lysine content and digestibility [59]. Considering the better bioactive profiles of colored rice, the contributions of these protein fractions are therefore attractive breeding objectives when developing varieties with superior nutritional profiles. Rice breeders from different countries are attempting to develop functional rice cultivars with desired glutelin levels; for example, a novel low-glutelin *indica* rice variety “Yishenxiangsimiao” with good quality and aroma has been developed in China [60]. There are currently multiple reports on the quantitative trait loci mapping for protein content in rice and fine location of the related genes is underway. However, detailed studies of types of rice proteins, their molecular structure, and how they interact with other nutritional components are limited [61]. Similar research in colored rice should be initiated and the existing knowledge be applied. Detailed comparative profiling related to protein and amino acids of purple, red, golden, and white rice grains has shown that purple rice is rich in L-alanine, L-arginine, L-asparagine, L-glutamic acid, L-methionine, L-ornithine, L-phenylalanine, and L-tryptophan [54]. Brown or red rice still provides higher essential amino acid content than white rice (Table 1) [54]. These comparative protein (and amino acid) profiles are important from practical applications as well. It has been found that lysine-rich rice can partially and positively affect the growth and development of the skeletal system in rats [62]. Therefore, increasing the colored rice intake, particularly of purple or red rice, would impart significant health benefits. Moreover, colored rice-based food can also be supplemented with a lysine-rich ingredient, e.g., red beans [63]. Several researchers are attempting to study the health-beneficial activities of pigmented rice proteins so that a useful recommendation can be made on the use of these rice varieties as functional foods. For example, a recent study measured the antioxidant, antidiabetic, and antihypertensive activities of defatted pigmented rice bran protein hydrolysates and indicated their utility as nutraceuticals [64]. However, recommendations on the amount of colored rice intake for individual nutrients are yet to be formulated based on the comparative nutrient profiles of different pigmented rice varieties, geographies, and types of disease or disorder.

Lipids are the third major component of rice gains, and are highly relevant to rice quality. Although the lipid content in rice is low, its composition, types of lipid species, and their richness contribute significantly to rice nutrition [65]. This is due to the fact that rice fat is higher in unsaturated fatty acids than saturated fatty acids (Table 1). Metabolome profiling of colored rice has identified lipids associated with multiple lipid biosynthesis and metabolism pathways, e.g., sphingolipid, linoleic acid, glycerophospholipid, glycerolipid, and ether lipid [54]. Studies on colored rice have reported the presence of mono-, di-, and tri-acylglycerols, free fatty acids, phytosterols, triterpene alcohols, tocopherols, and tocotrienols. Among fatty acids, palmitic acid, oleic acid, and linoleic acid have been mostly detected in rice samples [66]. However other fatty acids such as palmitoleic, stearic, arachidic, α-linolenic, behenic, and lignoceric acids have also been detected in black and red rice [35]. Generally, each type of colored rice differs in its fat content and composition; for example, red rice contains 1.15–3.19 g fat per 100 g [3]. Red rice (2.10%) has also been reported to contain the highest total % lipid compared to black (1.40%) and white rice (0.9%) [5]. With high white rice consumption associated with risks of type 2 diabetes, chronic diseases, cardiovascular diseases, etc. [67,68,69], the relatively lower carbohydrate and higher protein and lipid contents in specific pigmented rice varieties can provide a more balanced diet. In particular, the higher lipid contents of purple-black and red rice can positively impact health. Moreover, rice lipids, especially phospholipids, have been proposed as a way forward to improve rice gain quality [70]. It is common observation that brown and black rice have a higher fat/total lipid content than white rice (Table 1). However, studies have also reported variations, such that a comparison of black, red, and white Chinese rice has shown that crude fat content was higher in red rice, followed by white and black rice [54]. Moreover, black rice has also been reported to have higher total monounsaturated and polyunsaturated fatty acids than red and white rice [35]. The lipids in black rice consist of triglycerides, wherein glycerol is esterified with three fatty acids—oleic (42.10%), linoleic (29.30%), and palmitic acid (20.30%)—based on total lipid content. Nevertheless, the consumption of colored rice, whether red or black, offers a much better lipid content and should be considered a healthier option in line with The Dietary Guidelines for Americans, which indicates that less than 10% of calories a day should be from saturated fats (https://www.dietaryguidelines.gov/). It is also important to highlight that other than these fatty acids, rice also contains γ-oryzanols, which consist of ferulic acid esters from sterols and triterpene alcohols. Among its components, colored rice has higher contents of 24-methylenecycloartenol, cycloartenol, β-sitosterol, and campesterol [35]. The total γ-oryzanol content of both red (79 µg/g) and black rice (63 µg/g) is similar but significantly higher than that of white rice (8.2 µg/g) from Korea [35]. However, γ-oryzanol content can exceed these limits in rice. For example, black and red varieties from Taiwan contained up to 885 and 1727 µg/g of γ-oryzanol. Researchers have suggested the use of γ-oryzanol composition patterns as a basis for the classification of landraces. For example, a study using 223 Korean rice landraces, including five pigmented varieties, indicated that using γ-oryzanol individual component patterns for rice from different geographies and origins can be used to develop a model for varietal identification [71]. However, detailed work on the reliability of the information provided by γ-oryzanol is required since growth temperature [72], genotype by environment interactions [73], soil salinity [74], and other factors can influence its content.

**Table 1:** Basic nutritional profile of purple-black, white, and red-brown rice.

Nutrient	Purple-Black Rice	White Rice	Red-Brown Rice		References
			Brown Rice	Red Rice	
Energy/kcal	341	345–349	367	408.6	https://www.boohee.com https://www.foodwake.cn
Protein/g	9.4	7.7–7.9	7.54	9.1	
Carbohydrate/g	72.2	77.4–78.3	76.25	87.2	
Fat/g	2.5	0.6	3.2	2.6	
Dietary fiber/g	3.9	0.6–0.8	3.6	4.4	
Carbohydrate Profile					
Amylose (%)	1.9–13.8	14.3	2.8–25		[75,76,77]
Starch (%)	73.5–79.6	78.7	74.7–76.7		
Amylopectin	89.87	70–100	75		
Protein Profile					
Albumin (Alb) g/100 g	1.208	0.67–2.0	0.9–2.3		[78,79,80]
Globulin (Glo) g/100 g	0.327	0.652–2.0	0.67–2.3		
Prolamin (Pro) g/100 g	0.119	0.20–2.3	0.28–2.73		
Glutelin (Glu) g/100 g	7.929	1.684–5.258	2.0–6.18		
Amino acid Profile (ng/mg)					
beta-Alanine	611.72	1120.07	676.04		https://www.foodwake.cn; [54]
gamma-Aminobutyric acid	4924.82	24,773.64	56,672.55		
L-Alanine	50,803.14	30,287.82	43,769.23		
L-Arginine	39,126.49	22,084.74	15,158.64		
L-Asparagine	149,503.2	85,589.82	61,915.03		
L-Aspartic acid	122,281.8	167,794.52	80,174.63		
L-Glutamic acid	227,473.6	112,713.66	53,711.18		
L-Glutamine	23,746.48	98,415.37	86,887.77		
L-Histidine	4766.91	8966.76	20,289.02		
L-Isoleucine	18,844.8	31,887.29	30,977.34		
L-Leucine	2147.2	5393.29	6076.29		
L-Lysine	8572.17	11,177.98	16,866.32		
L-Methionine	1963.14	967.09	832.6		
L-Ornithine	1372.53	1024.82	938.33		
L-Phenylalanine	11,641.85	12,563.72	10,775.23		
L-Proline	17,804.19	10,371.11	20,058.6		
L-Serine	27,620.62	42,558.51	33,615.54		
L-Threonine	7630.53	15,770.47	19,250.02		
L-Tryptophan	45,506.56	13,171.08	24,495.74		
L-Tyrosine	1503.94	2772.80	5209.25		
L-Valine	7372.66	14,789.27	18,103.1		
Taurine	1208.35	800.74	1588.84		
Fatty Acids Profile (% of total fatty acids)					
Palmitic	19	21	20.96		[35,81,82]
Palmitoleic	0.21	0.27	0.18		
Stearic	2.5	3.76	2.80		
Oleic	40	39.19	38.60		
Linoleic	36	33.99	35.88		
Arachidic	0.6	0.76	0.54		
α-Linolenic	1.50	1.18	1.35		
Behenic	0.30	0.38	0.28		
Lignoceric	0.60	0.79	0.55		
Total lipids (%)	1.40	0.90	2.10		
Total γ-oryzanol µg/mg	63	79.00	8.2		

### 2.2. Minerals and Vitamins

Among other nutritional components, colored rice also contains minerals. Minerals in the human body mainly originate from the food we intake, and they play vital roles in the effective functioning of the body [83]. The human body requires at least 23 minerals, and most studies on rice, including colored rice, have reported 13 of them (Table 2). In addition, these elements also play essential roles in the growth and development of rice [84]. Although genetic characteristics, environmental conditions (especially the soil type), and nutritional profile are important characteristics influencing the rice mineral content, general trends in different colored rice varieties have been reported [85]. In colored rice, black rice is richer in essential minerals such as calcium, magnesium, phosphorus, zinc, sodium, potassium, sulfur, chlorine, and iron, whereas red-brown rice could be ranked second after black-purple rice regarding the mg/100 g content of these minerals. Moreover, red rice is richer in selenium, copper, and manganese (Table 2). Research on rice varieties with different characteristics, such as aromatic versus non-aromatic rice and glutinous versus non-glutinous rice, has revealed that rice varieties differ in their nutritional profiles [86,87,88]. For example, Indian brown rice offers a range of content of these minerals [3]. A study of the four types of Chinese colored rice, i.e., red, purple, golden, and white, showed that red had the highest iron content followed by purple, golden, and white [54]. Similarly, pigmented rice from Philippines showed higher zinc, iron, manganese, and phosphorus contents compared to non-pigmented rice [89].

Colored rice is also a rich source of vitamins, particularly vitamin E (tocopherols and tocotrienols; α, β, γ, and δ) and vitamin B (thiamine, riboflavin, niacin, choline, panathothenic acid, B6, biotin, and folic acid) [90]. Vitamins A, C, and D are not present in rice [91]. Comparative vitamin profiling of 58 black, red, and white Malaysian rice varieties showed that α-T and γ-T were the major tocopherols. Generally, black and white rice had higher quantities of α- and γ-tocopherols, whereas γ-T3 was the main tocotrienol, followed by α-T3 and δ-T3 [92]. The biosynthesis of tocopherols and tocotrienols can differ during growth stages of rice. Moreover, it may also be affected by the genetic differences and growth conditions. For example, different black and red rice varieties from Thailand showed no significant differences in tocopherol and tocotrienol contents [93], whereas black Malaysian rice was the richest source of vitamin E compared to red and white rice [92]. In the case of Taiwanese pigmented rice, the black variety had higher α, β, and δ tocopherols and α, β, γ, and δ tocotrienols compared to the red variety [94]. A comparative analysis of black-purple, brown-red, and white rice from Republic of Korea revealed that black rice had significantly higher γ-oryzanols, tocols (γ-T3, α-T3, and α-T), and total folate [35]. Taiwanese pigmented rice is higher in tocopherol and tocotrienol contents compared to that of Thailand and Malaysia. This could be due to genetic background, environmental conditions, and the extraction, detection, and quantification techniques [92,93,94]. Other than vitamin E, colored rice also contains vitamins K and B, although the research on the quantification of these vitamins is limited. Studies on the comparative nutritional profiles of black and white Indian rice varieties have shown that the former has higher thiamine (B1), riboflavin (B2), and niacin (B3) [95,96]. Vitamin B6 in Korean black rice was found to be 0.105 to 0.129 mg/100 g [97], which is higher than in white rice [98]. However, brown rice contains relatively higher B6 content (0.28 mg/100 g) compared to black rice.

Overall, the surveyed literature indicates that both purple-black and red rice are better sources of nutrients compared to white rice. In particular, the higher protein, dietary fiber, lipid, α- and γ-tocopherols, tocols, and folate contents in pigmented rice varieties make them nutritionally richer and healthier than white rice. However, recommendations on the use of a specific type of colored rice variety, under individual health conditions, are to be developed on individual basis.

**Table 2:** Nutrient and vitamin profile of black, white, and red-brown rice.

Minerals				
Nutrient/Vitamin	Black Rice	White Rice	Brown-Red Rice	References
Calcium/mg	12	11–12	9	https://www.foodwake.cn; [86,87,88,89]
Magnesium/mg	147	28–34	116	
Phosphorus/mg	356	112–121	311	
Zinc/mg	3.80	1.45–1.47	2.13	
Sodium/mg	7.1	1.7	5	
Potassium/mg	256	97–109	250	
Sulfur/mg	177.62	66–37-81.7	64.1749	
Chlorine/mg	10.95	2.62–3.7	7.71	
Iron	1.6	1.1–1.6	1.29	
Selenium/µg	3.2	1.99–2.5	17.1	
Copper/mg	0.15	0.19–0.29	0.302	
Manganese/mg	1.72	1.27–1.36	2.853	
Iodine/µg	NR	2.8	NR	
Vitamins				
Vitamin A	ND	ND	ND	https://www.foodwake.cn; [35,92,94,97,98,99]
Vitamin C	ND	ND	ND	
Vitamin D	ND	ND	ND	
Vitamin E	43.92	38.55	29.77	
α-tocopherol/mg/Kg	3.32–16.47 ^1^ 35.40–62.10 ^2^	2.56–4.90 ^1^ 23.27–54.56 ^2^	2.82–14.10 ^1^ 27.66–103.73 ^2^	
β-tocopherol/mg/Kg	0.35–1.09 ^1^ 2.43–2.68 ^2^	0.27–0.41 ^1^ 0.83–2.24 ^2^	0.34–0.89 ^1^ 0.92–3.26 ^2^	
γ-tocopherol/mg/Kg	3.20–7.69 ^1^ 10.55–39.22 ^2^	3.49–6.13 ^1^ 15.51–33.38 ^2^	1.43–7.00 6.09–37.68 ^2^	
δ-tocopherol/mg/Kg	0.28–0.66 ^1^ 1.10–3.27 ^2^	0.20–0.43 ^1^ 1.34–1.67 ^2^	0.18–0.54 ^1^ 0.26–3.48 ^2^	
α-tocotrienol/mg/Kg	1.63–6.22 ^1^ 12.52–43.56 ^2^	1.03–2.21 ^1^ 12.52–43.56 ^2^	1.50–7.95 ^1^ 8.44–67.01 ^2^	
β-tocotrienol/mg/Kg	ND ^1^ 0.48–0.73 ^2^	ND ^1^ 0.06 ^2^	ND ^1^ 0.25–5.74 ^2^	
γ-tocotrienol/mg/Kg	11.52–27.35 ^1^ 97.79–130.64 ^2^	11.29–23.67 ^1^ 75.98–120.26 ^2^	15.34–31.14 ^1^ 68.52–151.09 ^2^	
δ-tocotrienol/mg/Kg	0.65–1.90 ^1^ 1.68–2.16 ^2^	0.52–0.68 ^1^ 1.45–1.78 ^2^	0.44–1.51 ^1^ 1.34–3.54 ^2^	
Vitamin K/µg	NR	0.1	0.6	
Vitamin B1 (Thiamine)/mg	0.67	0.65–1.76	0.29–1.38	
Vitamin B2 (Riboflavin)/mg	0.04	0.02	0.04–0.3	
Vitamin B3 (Niacin)/mg	0.19	0.05	3.5–5.3	
Vitamin B6 (pyridoxine)	0.105–0.129	0.112	0.28	
Vitamin B7 (Biotin)	0.024	ND	ND	
Vitamin B9 (Folate)	0.0391	0.016	0.0329	

Note: the ^1^ and ^2^ superscripts for the quantity of tocopherols and tocotrienols represent two different studies from Malaysia [92] and Taiwan [94], respectively.

## 3. Phytochemistry of Colored Rice

The health benefits (see Section 4) of colored rice are mostly associated with the phytochemicals found in its bran or endosperm. In addition to fiber, fats, protein, carbohydrates, and vitamins, colored rice is also rich source of anthocyanins, anthocyanidins, proanthocyanins, flavonoids, phytosterols, phenolic acids, alkaloids, lignans and coumarins, nucleotides and derivatives, polyketides, quinones, and terpenoids [100]. Developments in metabolomics have significantly enhanced our understanding of the presence of these phytochemicals in rice varieties. This has been made possible because of improvements in analytical technologies such as gas chromatography–mass spectrometry (GC-MS), liquid chromatography–mass spectrometry (LC-MS), Fourier transform ion cyclotron resonance, and nuclear magnetic resonance. Concomitant developments in informatics techniques on the metabolomics data have also enabled researchers to conduct efficient peak picking, alignment, annotation, computational analyses, and enrichment analyses [101]. There have been several attempts to explore the metabolome profiles of brown, red, black rice, in comparison to white and green rice. Some studies also focus on green rice only [102]. Our review is focused on black-purple, brown, and red pigmented rice. However, to present logical comparisons and conclusions of the studies reviewed, we did not exclude green rice. Moreover, considering the fact that most studies performed comparative profiling, we present and discuss colored rice comparatively for global metabolome profiling, as well as individual metabolite class. In this regard, we surveyed published literature related to the terms “metabolomics”, “metabolome”, “global metabolome”, “comparative metabolome”, “secondary metabolites”, “pigmented rice”, “colored rice”, “black rice”, “purple rice”, “red rice”, “brown rice”, “pigmented rice comparison”, “secondary metabolites”, “flavonoids”, “non-volatile”, “compound classes”, and “anthocyanins”. The resulting literature was then manually reviewed, and only those studies were considered that reported comparative metabolomic or biochemical profiles of pigmented rice or in comparison to white rice. To find the literature containing quantitative data of each class of compounds, we also added the search term “quantification” with the names of each compound class. The literature that contained the content information was included and is discussed. Global metabolomics of pigmented rice has significantly expanded the number and range of detected compounds, such that some studies reported the detected more than 4500 ions. For example, comparative metabolome profiling of brown rice and white rice showed that, out of 4620 ions detected through LC-MS, 577 were differential. This study revealed that brown rice had higher flavonoids, fatty acids and conjugates, carboxylic acids and derivatives, organooxygen compounds, prenol lipids, and benzene and substitutive derivatives [100]. However, these profiles are not universal, as different varieties, even categorized under the same colored rice variety, can differ in composition and content of metabolites. Similarly, waxy and non-waxy pigmented rice varieties differ in their metabolite profiles. Among the Korean-bred waxy and non-waxy rice with a reddish pericarp, the latter has shown higher phytosterols (β-sitosterol, campesterol, and stigmasterol), whereas the waxy-type reddish rice had a higher content of phenolic compounds [103]. In most of the studies, black rice has been reported to have a higher-content nutritional profile than red-brown and white rice (Table 2). This is why black rice has been a subject of a higher number of metabolomic, biochemical, and health bioactivity-related studies. Moreover, metabolomic profiles of purple-black rice varieties from different countries have been reported, of which most focused on varieties and cultivars bred in and originating from Asian countries. Chinese purple-black rice had 390 differential metabolites compared to white glutinous rice; purple-black rice contains higher flavonoids, phenolics, and isoflavonoids compared to white rice [104]. When compared with white rice, brown rice had higher contents of compounds enriched in secondary metabolite pathways such as phenylalanine, tyrosine and tryptophan biosynthesis, histidine biosynthesis, purine metabolism, zeatin biosynthesis, and carbon metabolism [105]. Although most studies compare only one type of colored rice with white rice, there are also few studies including all categories of colored rice. A GC-flame ionization detector-based comparative metabolome analysis of red, black, and white rice revealed higher contents of fatty acid methyl esters, γ-oryzanols, anthocyanins, polyunsaturated fatty acids, triacylglycerols, folate, and phenolics in black rice, followed by red and white rice [35]. However, more sensitive techniques such as ultra-performance-LC tandem-MS have revealed a higher number of compounds present in colored rice. For example, a comparison of black, red, glutinous, and white rice reported the identification of 732 metabolites belonging to different compound classes [106]. A literature survey on the metabolome composition of colored rice indicates that different varieties differ within the same type of colored rice. Generally, flavonoids (16.67–18.86%) are the major compounds in colored rice, followed by lipids (12.86–17.62%), amino acids and derivatives (10.25–15.82%), phenolic acids (~14%), organic acids (7.14–10.52%), and alkaloids (9.23–10.25%), whereas quinones, tannins, and terpenoids account for ~1–3% each [104,106,107]. Apart from varietal differences, the metabolomic profiles and compositions can change if the rice is cooked with different methods [104,108,109]. Nevertheless, our literature survey highlights the limited availability of comparative metabolome profiling studies involving all types of colored rice from diverse geographies. This implies that such detailed studies must be conducted to understand varietal differences and the range of metabolites therein. Here, we briefly review the key metabolomic compound classes found in colored rice.

### 3.1. Flavonoids

Flavonoids, consisting of C6–C3–C6 rings, belong to the class of the polyphenolic group of secondary metabolites and are known for their beneficial impact on both the plants themselves and human health. In many nutraceutical, pharmacological, medical, and cosmetic uses, they are regarded as an essential component due to their ability to modify the activity of important cellular enzymes, as well as their antioxidant, anti-inflammatory, antimutagenic, and anticarcinogenic properties [110]. Colored rice is rich in flavonoids. Flavonoids in colored rice belong to sub-classes including anthocyanins, flavonoids, dihydroflavones, chalcones, flavanols, flavanones, flavonoid carbonosides, and flavanols [111]. Of these, flavonoids, flavonols, and flavonoid carbonosides are the major sub-classes in terms of the number of compounds detected from colored rice. Among colored rice varieties, black rice contains the highest content of anthocyanins, followed by flavonols, flavonoids, dihydroflavonols, flavonoid carbonosides, and chalcones, whereas red rice is richer in flavonoid content followed by flavonoid carbonosides, flavonols, flavanols, dihydroflavonols, chalcones, and anthocyanins [106,107,111]. This is probably because red rice contains proanthocyanidins instead of anthocyanins [28]. Nevertheless, the highest total anthocyanin content has been reported in black rice bran (294.62 mg Cy3-GE/100 g DM), followed by red (77.87 mg Cy3-GE/100 g DM) and brown rice (10.72 mg Cy3-GE/100 g DM). This signifies the importance of both purple-black and red rice in health-beneficial activity-related studies and their utility as functional foods. Significant variation exists regarding the content of total anthocyanins in colored rice. For example, black sweet rice from Canda had 3276 µg/g total anthocyanin content detected by high-performance liquid chromatography and LC-MS [1], whereas the Indonesian black rice variety Toraja had 21,120 µg/g anthocyanin content detected by the pH difference method [112]. However, such a large difference could be because of the sensitivity of different extraction, detection, and quantification techniques used. There is a limited number of studies that compare the total flavonoid, flavonoid composition, or anthocyanin content and composition using the same methodology. A recent review of the extraction, quantification, and characterization techniques for anthocyanin compounds indicated that different techniques yield different quantities in foods [113]. Thus, to receive reliable comparative data, it is recommended to use standard developed methodologies, or new standards should be established. Apart from the techniques, other factors such as the type of rice, i.e., waxy or non-waxy, genotype, production technology, climatic conditions, and environment can also influence their content and composition. This is somewhat evident from the summarized information on differences in flavonoid, anthocyanin, proanthocyanin, and phenolic contents in different colored rice varieties from different countries in Table 3. A study comparing Korean waxy and non-waxy red rice indicated that the former had higher quantities of flavone, flavonols, flavanone, and flavanol [103]. Similarly, high genotypic variation among the eleven Indian pigmented rice genotypes suggests that flavonoid content and composition should be carefully associated with a specific color rice type, although anthocyanins and proanthocyanins have been associated with types of pigments, and therefore the colored rice types. Moreover, a seasonal effect (environment) has also been reported to be a contributing factor [114]. Therefore, even with the same type of pigmented rice, the genotype, environment, and their interaction can influence the phytochemical contents, including of flavonoids, and their antioxidant capacities. For example, a study using 14 Chinese red rice varieties indicated that genotype x environmental effects were significant for total flavonoid content [115]. Thus, when formulating any nutritional recommendations, such knowledge can help in understanding the variation in flavonoid or other phytochemical contents. Nevertheless, flavonoids have been the most explored secondary metabolites in terms of their quantity and associated with health-beneficial activities in different colored rice varieties. More data indicate that black rice varieties are richer in flavonoid and anthocyanin contents than red and brown rice. Thus, among the colored rice types, black rice might offer better health benefits compared to other types [16,111,116]. However, several other considerations on the recommendation of each type of colored rice regarding flavonoid or anthocyanin intake should be considered. These include gender, age, ethnicity [117], geography [118], and dietary patterns (habitual flavonoid intake) [119]. Mean global flavonoid intake ranges from 150 to 600 mg per day. Thus, considering that some purple-black rice varieties may contain ~405 QE mg/100 g flavonoids [120], inclusion of 100 g of this type of colored rice can help fulfil the daily intake requirements. Regardless of the individual dietary requirements and health conditions, the daily intake of purple-black rice is a healthy choice [26].

**Table 3:** Phenolics, flavonoids, and anthocyanin contents and antioxidant activities of some colored rice varieties from different countries.

Phenolics	Flavonoids	Anthocyanin	Proanthocyanidin	Antioxidant Capacity	Cultivar	Country of Origin	Reference
Black-Purple Rice							
	7.6 mg/g FW	2.6 mg/g FW		~31.0 µmol Trolox/g ABTS value	Yangzi 6 hao	China	[19]
				~7.6 Trolox/g DDPH value			
				~5.8 Trolox/g FRAP value			
172 mg CE/100 g	125.60–155.45 mg CE/100 g			7.21 µm TE/g DPPH	Binhei and Heixiangdao	China	[121]
				4.83 µm TE/g ABTS	Binhei and Heixiangdao	China	[121]
95.33 µg/100 g	28 mg RE/100 g DW	51 mg CyGE/100 g		61.00%	Riceberry	Thailand	[122]
78.72 µg/100 g	29 mg RE/100 g DW	62 mg CyGE/100 g		63.00%	Hom Nil	Thailand	[122]
361–436 GAE mg/100 g	359–405 QE mg/100 g	189–239 C3G mg/100 g	76–146 CAE mg/100 g	35.14–41.25% Free Phenolics DPPH	Chakhao poireiton, Farmer collection	India	[120]
				65.92–75.14% Bound Phenolics DPPH			
				3–8 µM TE/g Free Phenolics ABTS			
				6–12 µM TE/g Bound Phenolics ABTS			
5267.20 µg/g		3276–5045.6 µg/g	32.10 µg/g	22 TAC, μmol/g	Minenomurasaki	Japan	[28]
Red Rice							
1.2 mg/g	2.0 mg/g	0.6 mg/g		6 µmol Trolox/g ABTS value	Hongnuo	China	[19]
				2.0 Trolox/g DDPH value			
				1.3 Trolox/g FRAP value			
61–68.06 mg CE/100 g	205.46–249.40 mg CE/100 g			4.28–6.26 µm TE/g DPPH	Caofeihong and Yanzhidao	China	[121]
				4.70–5.27 µm TE/g ABTS			
50.42 µg/100 g	20 mg RE/100 g DW	22 mg CyGE/100 g		60%	Mali Dang	Thailand	[122]
48.04 µg/100 g	16 mg RE/100 g DW	13 mg CyGE/100 g		41%	Mun Poo	Thailand	[122]
		93.5	3060.60	5	Yuyakemochi	Japan	[28]
215–439 GAE mg/100 g	258–399 QE mg/100 g	2.5–3.9 C3G mg/100 g	77–204 CAE mg/100 g	25.60–37.60% Free Phenolics DPPH	Ghaselu, Dumai, Kammra, Bakung, Maintinmolosty, Yangkum	India	[120]
				61.29–79.54% Bound Phenolics DPPH			
				0.16–1.3 µM TE/g Free Phenolics ABTS			
				2.4–3.1 µM TE/g Bound Phenolics ABTS			
White Rice							
0.5 mg/g	1.2 mg/g	<0.1 mg/g		7 µmol Trolox/g ABTS value	Yangchannuo 1 hao	China	[19]
				2.0 Trolox/g DDPH value			
				1.3 Trolox/g FRAP value			
45.75 µg/100 g	17 mg RE/100 g DW	ND		39%	KDML105	Thailand	[122]
59.58 µg/100 g	10 mg RE/100 g DW	ND		20%	Sung Yod	Thailand	[122]
59–80.39 mg CE/100 g	29.20–40.72 mg CE/100 g			1.95–2.85 µm TE/g DPPH	Nongken2021 and Zhongzao35	China	[121]
				2.53–2.87 µm TE/g ABTS	Nongken2021 and Zhongzao35	China	[121]
61–120 GAE mg/100 g	89–133 QE mg/100 g	0.4–2.98 C3G mg/100 g	0.1–0.44 CAE mg/100 g	19.40–48.21% Free Phenolics DPPH	PR I14, I21, I26, Basmati 370, Punjab Basmati 7, Pusa Basmati 1121	India	[120]
				38.11–70.98% Bound Phenolics DPPH			
				0.06–0.14 µM TE/g Free Phenolics ABTS			
				1.1–2.2 µM TE/g Bound Phenolics ABTS			

### 3.2. Lipids

The lipids in rice grains consist of triglycerides, phospholipids, fatty acids, and various bioactive lipid components, totaling 2–3%, significantly lower than that of oilseeds (Table 1) [123]. The metabolomic profiles of colored rice have revealed that lipids are the second most abundant compound class after flavonoids. Hundreds of metabolites classified as lipids and lipid-like molecules have been detected in colored rice. For example, a study involving red, black, white, and green rice detected 129 lipid compounds [106]. Another study using UHPLC-MS reported 69 lipid and lipid-like compounds (ceramides, diradylglycerols, triradylcglycerols, linoleic acid and derivatives, glycerophosphates, glycerophosphocholines, glycerophosphoethanolamines, and monoradyglycerols) [54]. Comparative metabolomic analyses have indicated that both oleic acid and linoleic acid are the major fatty acids in colored rice followed by palmitic acid, stearic acid, α-linoleic acid, arachidic acid, lignoceric acid, behenic acid, and palmitoleic acid [35]. Though limited in number, these studies have broadened the list of potential compounds to be further characterized in colored rice and to understand their individual or cumulative health effects. While lipid content in colored rice is evidently and understandably lower than in oilseeds [123], research on the development of mutants with a high lipid content (4.4% in Korean brown rice mutant P31-2-2-2-B-B) and the search for associated QTL is underway [124]. Such breeding efforts need to be expanded to both purple-black and red rice cultivars so that a more balanced nutritional profile can be achieved. Among the colored rice types, black rice contains higher contents of both oleic and linoleic acid (α- and γ-) than red or white rice. Other than these, octanoic acid, undecylic acid, dodecanoid (lauric) acid, myristoleic acid, pentadecanoic acid, 10-heptadecenoic acid, 9,12-octadecadien-6-ynoic acid, crepenynic acid, punicic acid, octadeca-11E,13E,15Z-trienoic acid, dihydroxypalmtic acid, 12-oxo-phytodienoic acid, ricinoleic acid, etc., have been detected and quantified in colored rice [106]. Of these, octanoic acid, lauric acid, dodecanoic acid, palmitic acid, and palmitoleic acid are present in colored rice in the least quantities. Moreover, several glycerol esters, lysophosphatidylcholine, and lysophosphatidylethanolamine have been identified and quantified from colored rice [106]. Studies on white rice have indicated that the native lipids, i.e., diacyl- and triacylglycerols, phosphatidylcholines, and lysophospholipids can affect starch digestibility differently [125]. However, similar knowledge on the starch digestibility under the influence of different lipid profiles of pigmented rice compounds is scarce. In this regard, readers are directed to a recently published review on the hydrolysis of starch in pigmented rice to understand the starch digestion aspects of pigmented rice [126]. Nevertheless, recent developments in metabolomics have revealed that, although in lesser quantities, the lipids in colored rice gains belong to diverse sub-classes and call for the application of other genomic and transcriptomic approaches, as well as GWAS to delineate the respective pathways. Such efforts may lead towards breeding colored rice cultivars with the required content and composition of lipids.

### 3.3. Amino Acids and Derivatives

Amino acids, in addition to being the structural unit of proteins, also act as metabolites and play roles in rice’s growth, development, and stress tolerance [127]. The composition and content of amino acids in rice grains vary widely. The amino acids in mature rice grains are protein-bound, and thus their content, distribution, and composition depend on the type of protein (albumin, globulin, prolamin, and glutenin) and its amount in rice [128]. The amino acids bound to albumin and globulin mostly accumulate in rice gaining parts other than the endosperm, whereas those bound to prolamin and glutenin are distributed in endosperm. Research on the amino acid profile of colored and white rice has revealed the detection and quantification of 20 amino acids [127,129]. As discussed in Section 2, different colored rice varieties differ in their storage protein contents; therefore, it is understandable that amino acid contents will also vary. Comparative metabolomic analysis of brown, white, and black rice from Korea revealed that black rice contains the highest content of alanine and aminobutryric acid. Moreover, glutamic and aspartic acids were the major amino acids, while phenylalanine, lysine, and histidine were present in minor quantities [129]. However, the list of the detected amino acids and their derivatives is expanding, thanks to developments in detection techniques. For example, more than 70 amino acids and derivatives have been detected and quantified in black, red, green, and white rice. Of these, notable are oxiglutathione, N-acetyle-, 5-hydroxy, 3-methyl-, N-monomethyl-, L-alanyl-, L-leucyl- dipeptides, S-allyl-, N-α-acetyl-, N-formyl-, N-monomethyl-, and other derivatives [106]. Additionally, the reduced forms of amino acids (e.g., glutathione), sulfoxides, and methyldopa have been detected from colored rice. Like the other metabolites discussed in the above sections, amino acid content and composition may differ in rice from different genetic and geographic backgrounds [130]. Chinese colored rice had highest contents of L-glutamine, L-lysine, L-phenylalanine, L-valine, and L-serine. Moreover, the study also revealed that red rice was richer in these amino acids compared to white and black rice [106]. Another UHPLC-MS-based metabolomic comparison of Chinese colored rice suggested that black rice was richer in L-arginine, L-asparagine, L-glutamine, L-methionine, and tryptophan [54]. Thus, both red and black rice are superior in terms of their amino acid profiles and contents than white rice.

### 3.4. Phenolic Acids

Phenolic acids are derivatives of cinnamic acid and benzoic acid. Rice accumulates both soluble (free as well as conjugated) and insoluble bound forms of phenolic acids. The phenolic acid fraction in rice includes a diverse range of compounds including hydroxybenzoic acids such as salicylic acid, 4-hydroxybenzoic acid, gentisic acid, protocatechuic acid, gallic acid, syringic acid, and vanillic acid. In addition, rice also contains hydroxycinnamic acids such as p-coumaric acid, caffeic acid, ferulic acid, isoferulic acid, sinapic acid, caffeoylquinic acid, p-coumaroylqunic acid, and feruloylquinic acid [131]. The phenolic acid fraction of rice, including colored rice, also includes flavonoids and anthocyanins; flavonoids with a *x*-phenyl-1,4-benzopyrone backbone are an important part of the polyphenolics of rice [110]. Of the colored rice varieties, red rice has been reported to have the highest total phenolics content (6.55–51.86 mg gallic acid equivalent/100 g), followed by black rice (4.31–36.52 mg gallic acid equivalent/100 g) and white rice (0.79–3.21 mg gallic acid equivalent/100 g) [132]. Similarly, the total phenolic acid content of red rice bran is highest, followed by black, green, and white rice [133]. When comparing waxy and non-waxy red rice, the former contained higher contents of hydroxybenzoic acid and hydroxycinnamic acid. By using more sensitive techniques such as ultra-high performance liquid chromatography–MS/MS, widely targeted metabolomics studies have expanded the list of compounds detected in colored rice. A recent comparative metabolomic profile study of white, black, red, and green rice detected 224 phenolic compounds, including 122 flavonoids: protocatechuic acid, 2,3-dihydroxybenzoic acid, 2,4-dihydroxybenzoic acid, gentisic acid, 4-nitrophenol, dibutyl phthalate, and vanillic acid. Both black and red rice contained the highest contents of these compounds compared to white and green rice [106]. This study also reported that some phenolic compounds are absent in black rice but present in red rice, e.g., benzamide and p-hydroxyphenyl acetic acid, whereas red rice lacked chlorogenic acid methyl ester. However, the molecular pathways leading to such differences are yet to be elucidated. Future studies employing co-joint transcriptome and metabolome approaches might enable researchers to associate key pathways and genes with differences in phenolic composition. Such studies are important since the major health-beneficial compounds reported from colored rice, i.e., anthocyanins and flavonoids, are phenolic compounds. Such knowledge would be useful to improve the phenolic content/composition of colored rice varieties to make them a better functional food. The relative content of the phenolic acids in colored rice could vary from variety to variety. For example, differently from the other reports reviewed above, the metabolomic profiling of a Chinese black rice variety Huamoxiang No. 3 revealed that vanillic acid, 1-O-gentisoyl-β-D-glucoside, protocatechuic acid-4-O-glucoside, vanilloloside, methyl cumulate, isovanillic acid, and protocatechuic acid, amongst others, were the major compounds [104]. Although the genotype, geography, environment, agronomic practices, etc., are the factors affecting these differences in the field, the processing of colored rice also can change the metabolomic composition of the rice as some compounds can degrade [100,104]. For example, black rice, when processed under different cooking modes, such as bottom heating, induction heating alone, or with pressure, can change flavonoid and anthocyanin (reduced) and phenolic acid (increased) contents [104]. In another study, processing of purple rice affected citric acid and isocyanate [134]. The extent of cooking, i.e., the duration of processing, also affects the texture and phenolic content of pigmented rice. For example, when processed by steam, the total phenolic and anthocyanin contents decreased compared to the rice cooked by the microwave method [135]. The literature survey clearly indicates the superiority of colored rice (purple-black and red) for their phenolic content over white rice.

### 3.5. Organic Acids and Alkaloids

Carbohydrates and organic acids are the key compounds that are associated with the sensory quality and flavor of foods [136]. Organic acids are the compounds with one or more carboxyl groups that are acidic in nature. Their content is not only important in terms of direct usage of colored rice, but also as a processed food, e.g., pigmented rice wine [137]. More than 70 different organic acids have been detected in white, black, red, and green rice. The major organic acids in colored rice are citric acid, 3-hydroxybutyric acid, malonic acid, isocitric acid, quinic acid, muconic acid, azelaic acid, and others [106]. Other than these, 1-methylpiperidine-2-carboxylic acid, L-pipecolic acid, malic acid, 2-picolinic acid, cis-aconitic acid, phenylpyruvic acid, quinaldic acid, tartaric acid, and many others have been reported to be higher in unprocessed black rice when compared to cooked rice [104]. While research on the detection and characterization of the organic acids in the colored rice continues, it is important to highlight that the comparative profiling with the same technique should be a way forward. Moreover, it is essential to follow the same set of experimental conditions, i.e., agronomy, light, temperature, humidity, and others, to critically compare the group of organic acids present in each type. The comparative studies on global metabolomics have been given the least consideration to these aspects. Thus, classifying colored rice in relation to the organic acids they contain becomes a challenging task. This is mainly because agronomic conditions and other factors can result in different metabolome profiles, such that derivatives of the same metabolite/compound could be biosynthesized under different conditions [138].

Rice accumulates a number of biologically active secondary metabolites, including various alkaloids [139]. Studies on different types of white rice have reported the detection of a variety of alkaloids such as indolamides, amides, nitrogen-containing heterocyclic rings, and polyamines [140,141]. In case of colored rice, alkaloids, phenolamines, isoquinoline alkaloids, quinoline alkaloids, and plumeranes have been detected and quantified [106,142]. Black and red rice have been reported to be richer in alkaloids such as glucosides, ergotamine, trigonelline, betaine, 1-methoxy-indole-3-acetamide, serotonin, indole-3-acetic acid, and spermine [106]. The variety of alkaloids detected in colored rice is higher than in white rice, indicating that the former type of rice offers better advantages both to the plants and consumers [106,141]. However, quantitative analysis of different colored rice varieties indicates that these secondary metabolites are affected by the genetic background of the variety, as well as the environment in which it is cultivated. For example, comparative analysis of green, red, black, and white rice has indicated the detection of 75 alkaloids [54], whereas another study reported 107 alkaloids [106]. However, since the detection is also dependent on the technique used, such variation can be observed in studies. For example, there are more than 520 alkaloids in brown rice [100], indicating that colored rice is richer in its diversity of alkaloids and intermediate compounds. From the user’s perspective, the surveyed literature on comparative profiles indicates the superiority of both black and red rice for alkaloid content and composition over white rice [106,142]. Though alkaloids offer beneficial pharmacological activities, their toxicities for humans are not to be neglected [143]. Research on colored rice has mostly focused on the beneficial health activities, e.g., antioxidant activity [144,145]. This could be because of the previous list of the least-available alkaloids in colored rice. However, as indicated above, the detection of >100 alkaloids in colored rice [106] would open avenues of future research for determining the beneficial or toxic effects of these compounds on human health. Nevertheless, the collective nutritional profile and the phytochemicals present in both black and red rice make them functional foods.

### 3.6. Other Metabolites

In addition to the above-mentioned compound classes, rice also contains a number of other secondary metabolites such as lignans, terpenoids, coumarins, quinones, amino acids, and polysaccharides. Among these, lignans and coumarins are produced as a part of the phenylpropanoid biosynthesis pathway. In colored rice, lignans and coumarins consist of ~1.5% of the total metabolome [106]. Comparative analysis of red, green, black, and white rice has shown that both red and black rice are richer in lignin and coumarin contents compared to white and green rice [107]. Among lignans and coumarins, the latter are present in higher quantities. The coumarins detected in colored rice include 6-methylcoumarin, sideretin, 1-methoxyphaseollin, and scopoletin-7-O-glucuronide, whereas the commonly detected lignans are trachelogenin, isohydroxymatairesinol, syringaresinol (and its glucosides), isolariciresinol, and (8′R,7′S)-(-)-8-hydroxy-α-conidendrin. Another study indicated that black rice had the highest contents of agrimonolide and demethylwedelolactone, whereas red and white rice contained the highest contents of O-feruloyl 3-hydroxycoumarin [139]. However, like other metabolites, the content of coumarins and lignans could be different in different cultivars categorized as the same color and species.

Rice, including colored rice, is rich in carbohydrates. In terms of metabolomic profiling of colored rice, saccharides constitute ~6.19% of all the compounds in colored rice [107]. Comparatively, black rice has been reported to have higher total sugar contents (140.25 mg/g) than white (128.72 mg/g) and red (116.02 mg/g) varieties [54]. The metabolomic composition of colored rice showed the presence of nearly 50–80 saccharides, with D-panose, galactinol, and D-sucrose as the most accumulated in black rice, followed by laminaran [54,86,103,107].

Nucleotides and their derivatives are associated with the central metabolism in plants, including rice, and can act as signaling molecules [146]. From a nutritional perspective, they are considered as semi-essential nutritional components, as our body is capable of producing them [147]. They usually accumulate more in seeds during development; hence, their composition and content can play an important role in both seed health and consumer nutrition [148,149]. The metabolomic composition of rice has revealed that nucleic acids and derivatives accumulated in seed are adenine, cytidine, adenosine, crotonoside, guanosine, inosine, N2, N2-dimethyguanosine, trans-zeatin N-glucoside, inosine 5′-monophosphate, 5′-deoxy-5′-(methylthio)adenosine, etc. [148]. Generally, colored rice is richer in nucleic acids and derivatives compared to white rice. The derivatives reported in colored and white rice include amines, monophosphates, diphosphates, glucosides, and ribonucleotides. The highest content of this class of compounds has been detected in black rice, followed by red and white rice. Black rice contains the highest content of 2′-deoxyinosine-5′-monophosphate [107]. Overall, ~50 compounds included in this class have been reported from comparative analysis of colored and white rice [54,86,103,106,107].

### 3.7. Volatile Aroma Compounds

Flavor and aroma are key rice characteristics and contribute significantly to consumers choice. More than 200 volatile compounds have been detected in rice, including hydrocarbons, aldehydes, alcohols, ketones, acids, esters, and heterocyclic compounds [150]. The number and quantity of compounds in each class and their respective quantities differ in different colored rice varieties, as well as within varieties. A study on two white, two red, and nineteen black Thai rice varieties by SHS-GC-MS analysis detected 48 volatile compounds. These compounds belonged to compound classes such as phenylacetaldehydes, aldehydes, alkaloids, ketones, fatty acids, hydrocarbons, and fatty esters. Both black and white aromatic Thai rice (having elevated levels of 2-acetyle-1-pyrroline, which gives an aromatic flavor [151]) had higher contents of these volatiles than red varieties [152]. The key aromatic compounds in colored rice are methyl acetate, 2-methylpropanal, 3-methylbutanal, 2-methylbutanal, petane-2,3-dione, pentanal, acetic acid, and formyl acetate. Another study involving black, green, purple, red, and yellow colored rice using GC-IMS indicated that aldehydes were the most prevalent flavor volatile substances, followed by ketones, alcohols, pyrazines, ethers, furans, pyrroles, and pyridines. Acetone, furfural-D, nonanal, and 2-methyl butanal have been found to be the most abundant volatile substances, accounting for up to 19.41%, 12.15%, 10.37%, and 9.52%, respectively. Black rice is richer in these substances, except nonanal [153]. In addition to the varietal and environmental factors, the volatilome composition of colored rice also differs before and after cooking. Nevertheless, aldehydes and aromatics are still the main (~80%) substances present in cooked black rice [154]. Some researchers have highlighted the utility of odor activity values of different odor active compounds for the discrimination of rice types, i.e., premium, waxy, and black rice [155]. Though the aroma of each rice variety is a unique characteristic defined by the composition of volatile aroma compounds, such studies have found the main contributors of overall aroma, e.g., 2-acetyl-1-pyrooline in black rice [155], However, like white rice, the aroma-related compounds in colored rice are correlated with the environmental factors of the rice growing sites [156]. Moreover, the volatile aroma compound composition of colored rice changes during cooking [154], fermentation [157], and wine making [137].

## 4. Biological Activities of Colored Rice

As presented in Section 2 and Section 3, colored rice contains a range of primary and secondary metabolites. Of these, many compounds such as anthocyanins, proanthocyanins, flavonoids, phenolic acids, vitamins, and minerals offer health benefits. Research on colored rice has revealed that it is richer in nutrients as well as bioactive compounds, and hence offers health-beneficial properties [19]. Here, we surveyed the literature on the biological activities and health-related studies on colored rice. For this, we used specific keywords to search the relevant literature from Google Scholar and PubMed, such as “colored rice”, “pigmented rice”, “black rice”, “purple rice”, “red rice”, and “brown rice”, in combination with keywords related to health and bioactivities. These keywords include “antioxidant”, “anti-inflammatory”, “antitumor”, “antiviral”, “antibacterial”, “antiaging”, “cryoprotection”, “hyperglycemic”, “neuroprotective”, “antifatigue”, “cardiovascular”, and “hyperlipidemic”. We did not focus on the mechanisms and pathways that are regulated under the influence of the health-beneficial compounds present in these types of rice because individual metabolite classes act differently. Explaining individual compound class and relevant mechanism in the experimental organism is beyond the scope of our literature review. Generally, colored rice varieties, i.e., black, red, and brown, offer greater biological activities compared to white rice.

### 4.1. Antioxidant Activity

Antioxidants are the organic molecules that protect cells in the human body from the oxidative damage caused by reactive oxygen species (ROS). The antioxidant compounds detected in colored rice range from vitamin E (tocopherols and tocotrienols; α, β, γ, and δ), anthocyanins, flavonoids, and phenolic acids [158]. Most of the studies on colored rice discuss the antioxidant activities in relation to free and bound phenolic acids, flavonoids, anthocyanins, procyanidins, minerals, amino acids, and oligosaccharides for colored rice varieties processed differently (milled, dried using far-infrared radiation, germinated, polished, and parboiled effects). The antioxidant activities of colored rice differ based on the variety and the cultivation conditions. However, generally, the black-purple and red rice have higher antioxidant activities than white rice (Table 3). The antioxidant activities are measured in terms of (2,2′-azino-bis-(3-ethylbenzothiazoline-6-sulfonic) acid (ABTS), 1,1-diphenyl-2-picrylhydrazil (DPPH), and ferric reducing antioxidant power (FRAP) values. The ABTS assay determines the scavenging ability of the antioxidants compared to Trolox [159]. The DPPH assay is a spectrophotometric assay and measures the DPPH scavenging ability of the antioxidants [160]. FRAP is used to quantify the ferric reducing antioxidant power of the samples [161]. Of colored rice varieties, black-purple rice has been shown to have highest ABTS, DPPH, and FRAP values, followed by red and white rice, indicating that the former offers higher health benefits. This is true for a large number of colored rice varieties from China, India, Japan, Canada, and Indonesia [19,28,112,120,121,122,162]. Studies on colored rice have shown a similar pattern of antioxidant capacity [163]. However, the key question to be answered is which compounds (or classes) are linked with the higher antioxidant activities. A correlation analysis of the flavonoid metabolites and the antioxidant activity indicators revealed that 36 metabolites (mostly anthocyanins and flavonoids) are strongly and significantly correlated with ABTS, FRAP, and DPPH activities [19]. Since anthocyanins are mostly the pigments in colored rice that give distinct black-purple or brown-red colors, some researchers have positively correlated the color of rice with the DPPH and ABTS activities [128], although flavonoids are not the only compounds contributing to the antioxidant activities. Tocopherol and tocotrienol have also been tested for their role as antioxidant agents [92]. Notably, this study concluded that the observed antioxidant activity of pigmented rice was not solely linked with tocopherol and tocotrienol, but other bioactive compounds might also play strong roles as antioxidants. Hence, some studies indicate that when interpreting antioxidant capacity assays in complex systems, care must be taken to link tocotrienols [164]. Total phenolic content and proanthocyanidin content also exhibit a higher degree of association with the radical scavenging ability and DPPH values in pigment rice compared to non-pigmented rice [128,132,165]. However, the antioxidant activity of the soluble-free and soluble-conjugated phenolic fractions is significantly higher than the insoluble ones [132]. The bound and free forms of phenolics also exhibit different activities, such that the former has higher DPPH and ABTS activity [120]. Similarly, colored rice with higher anthocyanin and α- and γ-tocopherols also offers higher antioxidant potential in FRAP [166]. Higher antioxidant activity of black-purple rice has also been reported in a clinical trial. The trial reported that purple rice could increase antioxidant activity by 70.5% compared to red rice (21%) [167]. Nevertheless, since colored rice is not consumed raw but cooked, the polyphenol contents (phenolics and flavonoids) are reduced during such processing, and therefore the antioxidant activity is affected. However, during in vitro digestion trials, this reduction in flavonoids and phenolics during cooking rebounded during gastric and intestinal digestion phases [121]. Germinated colored rice is also considered a healthy alternative to white rice due to its physiological health benefits [168]. This is because germinated colored rice, e.g., brown rice, contains relatively larger quantities of bioactive compounds compared with non-germinated rice [169]. Tests on menopausal rat models have revealed that the consumption of germinated Superjami colored rice with a deep-violet pericarp results in higher activities of antioxidant enzymes compared to non-germinated and control diets [170]. Supplementation of colored rice can improve the antioxidant status of human and animals. This has been proven for healthy subjects after acute intake [171], patients with coronary heart disease [172], type 2 diabetic rats [173], an obese cohort [174], and atherosclerotic plaque rabbits [175,176]. Therefore, supplementation of colored rice in food can increase the antioxidant activity owing to the higher content of tocopherols, tocotrienols, vitamin E, flavonoids, anthocyanins, and phenols.

### 4.2. Anti-Inflammatory Activity

Inflammation is one of the body’s defenses by which the immune system recognizes and ultimately eliminates harmful stimuli after an injury. It consists of reactions at the cellular and microvascular levels and serves to eliminate damage and generate new tissues [177]. To be classified as an anti-inflammatory compound or drug, a substance should be able to inhibit inflammation without compromising homeostasis [178]. Colored rice contains several compounds (or classes) with anti-inflammatory activities. Natural rice bran oils from Thai colored rice contained higher contents of δ, γ, and α-tocotrienol, δ, β, γ, and α-tocopherol, and γ-oryzanol. The higher contents of these compounds were associated with anti-inflammatory activities compared to white rice in RAW264.7 mouse macrophage cells [179]. The suppression of nitric oxide, inducible nitric oxide synthase, and cyclooxygenase-2 in these mouse cells is concentration-dependent [180]. Among the bioactive compounds, γ-oryzanol from purple glutinous Thai rice was able to inhibit nitric oxide production [181]. However, there are other compounds in pigmented rice that also inhibit cellular nitric oxide and inducible nitric oxide synthase. For example, a study reported that such inhibitory activity was positively correlated with total phenolic content and flavonoid content [182]. Whether the colored rice is consumed by cooking or as a by-product, its anti-inflammatory effects are better than those of white rice and cow’s milk. For example, pigmented rice-milk kefir offers higher anti-inflammatory activity determined by blood chemistry, hematological evaluation, and tumor necrosis factor-a levels [183]. The anthocyanins present in purple-black rice have also shown anti-inflammatory effects. However, during oral consumption of colored rice, these compounds may be metabolized into respective derivatives or other compounds. For example, cyanidin-3-O-β-D-glycoside might change into cyanidin or protocatechuic acid. These compounds have the ability to reduce carrageenan-induced inflammation in mice by suppressing the production of proinflammatory and inflammatory mediators [184]. From the industrial usage point of view, it must be noted that raw rice usually has a higher level of bioactive compounds and, therefore, offers higher anti-inflammatory activity compared to processed rice; nevertheless, colored rice is far better than white rice [185]. Therefore, processing of colored rice should be considered carefully to avail higher health benefits [186]. The extracts from colored rice exhibit anti-inflammatory activity both in vivo [184] and ex vivo [187]. Among colored rice varieties, most studies have reported that black rice offers higher anti-inflammatory activity compared to brown-red and white rice. This can be because of the higher contents of bioactive compounds in the former. The bioactivities also depend on the variety and the content of bioactive compounds, especially flavonoids, phenols, and anthocyanins. A homogenous mutant AM22 of the Heishuai black rice variety contained less vitamin B2, flavonoids, and anthocyanins, and therefore, the anti-inflammatory activity was attenuated [188]. Therefore, supplementation of colored rice can significantly improve health in patients. For example, consumption of 10 g of black rice for six months in patients with coronary heart disease greatly increased antioxidant activities compared to a placebo group [172]. Clinical trials on an obese cohort of sedentary patients who consumed one cup of red, purple, and brown rice indicated that purple rice offered significantly higher anti-inflammatory activities after 30 min and one hour of consumption [167].

### 4.3. Antitumor (Anticancer) Activity

The antitumor activity of any substance refers to innate/adaptive immune responses that ultimately enable the cells/tissues to control the tumor, in any chemical substance that prevents the growth of cancerous cells. This is achieved by the death of abnormal cells that undergo uncontrolled division [189]. Rice cell suspension culture has been previously implicated in anticancer activities without causing any significant damage to normal cells’ growth [190]. Considering this, together with the fact that it is richer in secondary metabolite content and composition, colored rice should also offer antitumor activities. In particular, bioactive substances such as anthocyanins, flavonoids, phenolics, tocotrienols, tocopherols, and γ-oryzanol have been widely analyzed for their antitumor activities. Early research has indicated that anthocyanins (of red rice) offer tumor-suppressive effects in Balb/C mice and those inoculated with syngeneic meth/A lymphoma cells [191]. Anthocyanins from purple-red rice can significantly decrease spleen TNF-α, IL-6, and IL-1β levels [192]. Within colored rice varieties, our literature review has revealed the higher flavonoids, phenolics, and anthocyanins in black rice, followed by red and white rice (Table 3). In this regard, methanol extracts from the hull of black rice proved to be the most potent against human breast, melanoma, and oral cancers. This cytotoxicity has been associated with the ability of the extracts to induce apoptosis and DNA fragmentation [193]. Other mechanisms of this cytotoxicity are the growth inhibition of immune checkpoint inhibitors [194], and mortalin-p53 complex inhibition [195]. Since germinated rice has also been implicated in health-beneficial activities, the germinated colored rice can offer better health-promoting and antiproliferative effects [169]. For example, the ethanol extracts from germinated black-purplish and brown rice were higher than the extracts of non-germinated rice. However, the extracts from individual rice varieties of the same color could also differ in their activities. Extracts from the same variety might not be effective against all types of cancers. For example, the extracts from germinated black rice had higher anticancer activities on cervical and gastric cell lines, but lower activities in liver and colon cancer cells [196]. It is also to be noted that, in addition to the variety and type of colored rice, the fractions of extraction via different solvents may also impact anticancer activities [197]. Within the extracts, the key compounds associated with anticancer activity are phytosterols and triterpenoids [198]. Considering the antitumor activities of colored rice, efforts should be made to develop pharmaceutical products and nanoparticles [199]. These could be developed from the hull, bran, or the whole seeds [200].

### 4.4. Antimicrobial (Antibacterial and Antiviral) Activities

Bacteria and viruses, among other microbes, are responsible for a wide range of diseases and illnesses in humans [201]. Research on the antimicrobial activity of colored rice is an active topic and hundreds of studies have been undertaken to understand how colored rice consumption can offer suppressive activities against a diverse range of bacterial and viral genera/species. Colored rice, being rich in secondary metabolites, can significantly suppress bacterial and viral growth. Ferulic acid from Indonesian purple rice proved to be a strong antibacterial agent against *Salmonella typhimurium* and *Listeria monocytogenes*. It induced shrinkage and osmotic lysis of bacteria and inhibited binding of bacterial virulence factors with toll-like receptors [202]. Generally, methanol extracts of black rice have been proven to have antimicrobial activities against both Gram-positive and Gram-negative bacterial species [203]. Ethanol extracts of black rice can also inhibit the growth of oral bacterial species such as *Streptococcus mutans* and *Porphyromona gingivalis* [204], indicating that direct consumption could be beneficial. Black rice has also been proposed to manage gastroduodenal diseases caused by *Helicobacter pylori*, owing to the ability of cyanidin 3-O-glucoside’s inhibitory activity, again in the bacterial toxin section [205]. Due to the fact that black rice is richest among colored rice varieties in ferulic acid, oryzanol, phenolics, and especially anthocyanins, antibacterial gels have been successfully made. The gel has been tested against *Staphylococcus aureus* and *Escherichia coli* [206]. In addition to gel, black rice sourdough fermented with lactic acid bacteria has also been proven to have antibiotic activities owing to the organic acids [207]. The isolates of lactic acid bacteria from Indonesian fermented black rice (*Tapai*) significantly inhibited the growth in several bacterial species such as *E. coli*, *Pseudomonas aeruginosa, Bacillus subtilis, Kocuria rhizophilla,* and *S. aureus* [208]. Overall, these antibacterial activities are correlated with the phenolic, flavonoid, and anthocyanin contents [182].

Dietary flavonoids, phenolics, tocotrienols, tocopherols, and γ-oryzanol from food sources have been associated with a variety of biological activities, including antiviral activities. These compounds inhibit viruses by the inhibition of neuraminidase, protease, and nucleic acid polymerases and, in some cases, they are able to modify viral proteins [209,210,211,212]. Comparative antiviral activities of colored rice have not been extensively studied. Instead, individual varieties or extracts of colored rice have been reported to have antiviral properties. Since the major bioactive compounds in colored rice, especially black and red rice, are anthocyanins, most research has focused on their antiviral activities. Cyanidin-3-O-glucoside and peonidin-3-O-glucoside have been shown to inhibit the spike glycoprotein S1 subunit of SARS-CoV-2 [213]. Based on this understanding, black rice germ and bran have been proposed for consumption for long-term prevention of COVID-19. Anthocyanin extract from red rice is able to inhibit herpes simplex virus type 1 before, during, and after infection in Vero cell lines [214,215].

### 4.5. Hypoglycemic Activities

Hyperglycemia is a condition in which a patient has blood glucose levels higher than 125 mg/dL and 180 mg/dL during fasting and postprandial states, respectively. It is also considered as a risk factor for cardiovascular diseases [216]. Although rice, including colored varieties, has higher carbohydrate and starch contents, there is a wide range of secondary metabolites that offer antidiabetic activities [217]; in particular, the anthocyanins from colored rice have been associated with antidiabetic activities. Java red rice from Indonesia has been shown to inhibit α-amylase [218], which is a drug target to prevent postprandial hyperglycemia [219]. Replacing white rice with colored rice (brown, red, and black) can be an effective strategy to lead to hypoglycemia in hyperglycemic patients. In this regard, supplementation of black rice extract in a streptozotocin-induced type 1 diabetic rat model significantly reduced uric acid, aminotransferases, and alkaline phosphatase. This effect was also noted for brown as well as red rice supplementation [220]. Among the secondary metabolites, researchers have focused on cyanidin-3-O-glucoside and other compounds. The freeze-dried powder from a higher cyanidin-3-O-glucoside-containing black rice variety showed a significant reduction in the blood glucose level and inhibition of oxidative stress in blood [221]. Similarly, the ethanol extracts of brown rice were able to inhibit alpha-glucosidase activity [222]. The key metabolites that are involved in the inhibition of carbohydrate-hydrolyzing enzymes are vanillic acid, procyanidins, anthocyanins, phenolic acids, and p-coumaric acid [223]. Research has suggested that in addition to inhibiting enzymes associated with hyperglycemia, red and purple rice extracts can increase expression of genes involved in the insulin-signaling pathway [224]. In addition to anthocyanins, oryzanol, when orally administered to rats, can reduce blood glucose levels [225]. Since varieties differ in their metabolomic content and composition, specific regions can adapt selected varieties, and validate the amount of supplementation in normal and hyperglycemic people’s diets. Moreover, since processing also can affect the content of the secondary metabolites, the recommendations on the processed of colored rice for individual cases should be carefully developed [223].

### 4.6. Anti-Aging Properties

Aging is a natural phenomenon often associated with age-related diseases that place an economic burden on governments. The identification and use of functional foods in relation to anti-aging activities has been a major focus of researchers [226]. There are groups of both genetic and stochastic scientists who propose aging mechanisms, such as programmed theories, stochastic theories, the free radical theory of aging, energy restriction and lifespan, and the cross-linking theory of aging [227]. Since colored rice has been associated with antioxidative properties, they might also offer anti-aging benefits upon consumption. In this regard, limited work has been done. Nevertheless, the extracts of colored rice have been shown to play an anti-aging role. Treatment of stress-induced premature senescence of WI-38 human diploid fibroblast cells with the methanol extract of black rice could attenuate oxidative stress, improve their viability, and inhibit lipid peroxidation [228]. Ferulic acid from Indonesian purple rice can inhibit collagenase and tyrosinase activities, and therefore, has been proposed as an anti-aging candidate [229]. In addition to ferulic acid, anthocyanins from purple glutinous rice extract have also been implicated in anti-aging properties due to their inhibitory effect on tyrosinase enzymes, lipid peroxidation, and radical scavenging activity [230]. However, as indicated above, red rice often has a limited content of anthocyanins and is richer in proanthocyanidins, with some researchers focused on the anti-aging properties of these compounds. The activities of the skin aging enzymes, e.g., collagenase and matrixmetalloproteinase-2 are significantly inhibited by the main bioactive compounds of red rice, i.e., proanthocyanidins, catechins, hydroxybenzoic acid, vanillic acid, and oryzanol [231]. These studies indicate the positive impact of several compounds on skin, thus providing an opportunity for development of cosmetic products [232,233]. However, the anti-aging effects of the extracts of colored rice are concentration-dependent [234]. Thus, both the supplementation of colored rice in diets and their applications to skin can be beneficial [235,236].

### 4.7. Neuroprotective Properties

Owing to the increase in the prevalence of neurodegenerative disorders, the search for neuroprotective nutritional compounds is an active discipline. To be considered as a functional food against neurodegenerative disorders, the nutritional components must have the ability to activate receptors, modify neuroprotective enzyme activities, and play a role in the synthesis of molecules involved in alleviating cell loss or neural damage [237]. Foods rich in flavonoids, phenolic acids, and anthocyanins have been intensively studied for their neuroprotective roles [238,239,240]. Since colored rice is rich in these compounds, the neuroprotective impacts of colored rice have been studied. Among neurodegenerative diseases, Alzheimer’s disease is prevalent in humans. Most research on the effects of colored rice has been conducted related to this disease. Purple rice berry extract had a significant preventive effect on memory impairment and neurodegeneration in the hippocampus of the Wistar rats [241]. This activity is because of the protective ability of cyanidin from the cytotoxic effect of Aβ_25–35_-induced neuronal cell death in SK-N-SH cells. The anthocyanin component of the colored rice can significantly attenuate ROS and reactive nitrogen species [242]. In addition to anthocyanins, ferulic acid from red and black rice also offers a neuroprotective effect, as noticed in SH-SY5Y cells [243]. Colored rice extracts also offer neuroprotective properties in other neurodegenerative diseases such as Parkinson’s-like disease [244,245], ischemic brain injury [246], CoCl_2_-induced injury [247], and scopolamine-induced memory deficit [248]. Moreover, anthocyanins also offer neuroprotective effects on apoptosis [249]. Research has shown that both pre-germinated [250] and germinated colored rice [251] have neuroprotective effects. Thus, consumption of colored rice can offer neuroprotective benefits owing to the higher relative contents of anthocyanins, ferulic acid, flavonoids, and other bioactive compounds [252].

### 4.8. Clinical Trials

The literature surveyed and discussed above highlights that colored rice varieties offer a range of bioactivities and protective effects. Although such a vast amount of information is available on the utility of extracts from colored rice, including pre-cooked, cooked, and germinated, few clinical trials have been conducted. Recent research, as reviewed by several authors, has highlighted that pigmented rice consumption reduces cardiometabolic risks by increasing plasma antioxidant activity and reduction in postprandial glucose and insulin levels [11]. Authors of these reviews have indicated that trials comparing the consumption of pigmented rice, e.g., brown rice, with white rice have led to inconsistent findings. However, generally, brown rice, especially pre-germinated varieties, offers better functional properties by promoting lipid profile and fasting blood glucose levels [253]. Similarly, meta-analysis of randomized control trials assessing the efficacy of red yeast rice extract on myocardial infraction patients with borderline hypercholesterolemia have shown that consumption of 1200 mg/day has been proven to reduce nonfatal myocardial infarction. Similarly, supplementation of red yeast rice offers beneficial changes in lipid profile, i.e., lowers LDL, TC, and TG, while increasing HDL levels. A key feature of such studies is that the experimental duration is not consistent, i.e., 4 weeks to 4.5 years [254,255,256]. We further searched Google Scholar, Web of Science, and PubMed databases using keywords, i.e., pigmented rice, colored rice, and clinical trials, which yielded very limited results. For searching, we used different keyword combinations, e.g., colored rice, pigmented rice, black rice, purple rice, brown rice, and clinical trial, in different combinations. In the case of searching Web of Science and PubMed, the use of a simple combination of terms, i.e., colored rice + clinical trials or pigmented rice + clinical trials, resulted in three to five studies. Most of the literature surveyed was available from Google Scholar. However, the available literature on Google Scholar is highly variable, such that the articles that were viewed as a search result mostly contained text where the term “clinical trials” was used as a suggestion for future studies. Nevertheless, a key feature we observed in these meta-analysis studies, and research conducted by us, is that these clinical trials or intervention studies do not compare different pigmented rice varieties, e.g., brown, black-purple, and red rice, within one study. Moreover, the study designs are highly variable, therefore producing inconsistent results. However, these trails clearly highlight that the consumption of pigmented rice in any form is beneficial for health. Moreover, their consumption in both males and females, and participants with certain health conditions, i.e., obese menopausal women, those suffering from metabolic syndrome, having type 2 diabetes mellites, moderately hypercholesterolemic, with mil dyslipidemia, prediabetic, etc., is mostly beneficial (Table 4). The experimental duration is highly variable, i.e., ranging from minutes to months. However, detailed clinical trials in future should focus on standardized dietary interventions, dose, period of trial, and types of resulting parameters to be observed for each disease or health condition.

**Table 4:** Examples of clinical trials or dietary intervention studies conducted on the health benefits of colored rice.

Country	Study Design	Number Completed	Sex	Age	Health Status	Intervention	Dose	Experimental Duration	Outcome	Reference
Italy	Crossover randomized controlled clinical trial	19	Male and Female	24.7 ± 3.8	Healthy	Black rice	100 g serving	30, 60, 120, and 180 min	↑Plasma total phenol index Venera had higher than Artemide. ↑Total flavonoids ↑DPPH ↑ABTS	[171]
Korea	Placebo-controlled preliminary clinical trial	86	Female	56.91 ± 5.71 57.32 ± 5.45	Obese postmenopausal women	Black rice extract	1 g capsules (twice a day)	12 weeks	↓Trunk fat, ↓total fat, ↓total body fat % Non-significant differences in anthropometric measures	[257]
Indonesia	Clinical trail		Female	18–25	Health	Black rice bran extracts	0.5 g 10% black rice extract containing lotion—twice daily	14 days	↓Melatonin index. ↓Erythema index	[258]
Iran	Open label randomized controlled trial	50	Male	65–74	Suffering from metabolic syndrome	Brown rice bran powder	15 g brown rice bran powder	8 weeks	↓BMI ↓Waist circumference, ↓Total cholesterol ↓Blood sugar ↓Triglyceride glucose-BMI index ↑Antioxidant enzyme activities	[259]
Thailand	Intervention trial	120	Male	65–74	Healthy	Black rice germ and bran supplement	10 g black rice powder + exercise	24 weeks	↓Inflammatory biomarkers (c-reactive protein and interleukin-6 levels) ↑Insuline-like growth factor-1 level	[260]
Thailand	Randomized placebo-controlled clinical trial	60	Male and female		Type 2 diabetes mellitus	Dietary fibre (from Jerusalem artichoke and white rice bran) and anthocyanin (from riceberry rice)	1.26 g/day of each dietary fibre (from Jerusalem artichoke and white rice bran) and 0.28 g/day of anthocyanin (from riceberry rice)	60 days	↓Glucose and lipid profiles ↑Kidney’s function glomerular filtration rate	[261]
Japan	Open Label Test	65	Male and female	23.8 ± 8.8 22.0 ± 1.2 (CK)	Healthy	De-waxed brown rice vs. polished rice	>150 g per day, once a day	One month	Improved skin age, Improve wrinkles and porphyrin levels in Female subjects	[262]
Italy	Placebo-controlled preliminary clinical trial	90		30–75	Moderately hypercholesterolemic	Combined nutraceutical (phytosterols (800 mg) and red yeast rice)	One tablet after dinner (phytoesterols 800 mg + red yeast rice having 5 mg monacolins + niacin 27 mg + policosanols 10 mg)	8 weeks	Reduced the plasma levels of LDL-C, TC, non-HDL-C, ApoB, TC/HDL-C and LDL-C/HDL-C ratios in mildly hypercholesterolemic patients	[263]
Japan	Multi-centre randomized trial	18	Male and female	20–90	Mild dyslipidaemia	Red yeast rice	200 mg/day red yeastrice, containing 2 mg monacolin K, once a day with water after dinner	8 Weeks	↓Low-density lipoprotein cholesterol, ↓Total cholesterol, ↓Apolipoprotein B, ↓Blood pressure	[264]
India	Dietary-intervention	40	Female	25–50	Prediabetic women	Germinated brown rice	120 g daily	120 days	↓Fasting blood glucose, ↓average glycated hemoglobin, ↓total cholesterol, ↓Triglycerides, ↓Low-density lipoprotein cholesterol	[265]
Thailand	Randomized crossover	19	Male and female	18–40	Healthy	Anthocyanins from Riceberry rice	350 g whole milk, skimmed milk powder (3% *w/w*), and sucrose (5% *w/w*), with or without the anthocyanin-rich extract of riceberry rice (0.25% *w/w*).	15, 30, 60, 90, 120, 150, and 180 min	↑Plasma ferric reducing ability of plasma, Trolox ↑equivalent antioxidant capacity, ↑oxygen radical absorbance capacity, ↓Decrease in iAUC for plasma malondialdehyde	[266]
Thailand	Randomized control trial	24	Not specified	45–70	Healthy	Germinated Black Rice	1000 mg/day	8 Weeks	Improved working memory, (Decreased response time, attentional control, memory speed) ↓Acetylcholinesterase, ↓Monoamine oxidase	[267]
Chinese	Randomized control trial	24	Not specified	21–65	Healthy	Black rice anthocyanin extract	2% and 4% Black rice extract per 100 g of wheat flour	2–4 h	Improved plasma HDL-c, Apo-A1, Apo-B, modified LDL and HDL subfractions, remodelled lipid distributions in HDL and LDL particles	[268]

DPPH (2,2-diphenyl-1-picrylhydrazyl), ABTS (2,2′-azino-bis(3-ethylbenzothiazoline-6-sulfonic acid), BMI (body mass index), LDL-C (low-density lipoprotein cholesterol), TC (total cholesterol), non-HDL-C (non-high-density lipoprotein cholesterol), ApoB (Apolipoprotein B), TC/HDL (total cholesterol to high-density lipoprotein cholesterol ratio), iAUC (incremental area under the curve), Apo-A1 (Apolipoprotein A1), Apo-B (ApolipoproteinB), an upward arrow indicates an increase, and a downward arrow indicates a decrease.

Taken together, our literature survey indicates that almost all the literature surveyed regarding the health-beneficial activities of bioactive compounds in colored rice used methodologies involving in vitro assays. Only a limited quantity of clinical trial-based data on the use of pigmented rice is available. To achieve the maximum utility of the nutritional and bioactive profiles of pigmented rice and implement the knowledge in clinical practice, future studies must focus on prolonged in vivo studies and clinical trials. We say this because the currently available literature indicates that the clinical trials were conducted only for a short time, i.e., 30–180 min [171] and 1–4 h [167]. In contrast, some studies examined the effect of pigmented rice supplementation for several months, e.g., for 12 weeks in obese postmenopausal women [257], for 16 weeks in type 2 diabetes mellitus patients [269], and for six months in patients with coronary heart disease [172]. A key observation in these studies is that they mainly focus only one type of pigmented rice; hence, conclusions about the comparative clinical significance of different colored rice varieties cannot be drawn. Moreover, each study uses a different number of cohorts and different doses, concentrations, and form of pigmented rice (cooked, extract, or boiled). Thus, future studies must focus on long-term clinical trials on the use of pigmented rice, including different types of colored rice varieties with similar doses. Moreover, such studies must optimize and adapt standardized methodologies for measuring the biological activities. While choosing the most appropriate pigmented rice varieties, the effect of the genotype, environment, and their interaction should not be ignored. Such detailed long-term clinical trials would reveal useful data for applications in clinical practice.

## 5. Conclusions

Colored rice varieties—black, brown, and red—offer substantial nutritional advantages over white rice, primarily due to their unique nutrient and metabolome (volatile and non-volatile) composition. Black rice is rich in anthocyanins, especially cyanidin-3-glucoside, while brown rice contains proanthocyanins and red rice features a combination of both. These rice types also contain small amounts of carotenoids like lutein and zeaxanthin. These pigmented rice types generally provide higher energy values than white rice, with black and brown rice containing more amylose. They also have a lower glycemic index, making them healthier alternatives to white rice. Black and red rice are particularly rich in proteins, with glutelin being the primary protein, followed by albumin, globulin, and prolamin. Glutelins, known for their high lysine content and digestibility, are especially valuable, and research is focused on developing varieties with higher glutelin content. Additionally, black rice is abundant in essential amino acids, such as L-alanine, L-arginine, and L-tryptophan. Beyond proteins, pigmented rice varieties contain lipids, including free fatty acids, phytosterols, tocopherols, and tocotrienols. Fatty acids like oleic acid, linoleic acid, and palmitic acid are commonly found in brown and black rice, which have a higher lipid content than white rice. Additionally, colored rice contains γ-oryzanols, including 24-methylenecycloartenol and β-sitosterol, which contribute to a healthier lipid profile in line with dietary guidelines for limiting saturated fat intake. Minerally, black rice stands out as a rich source of calcium, magnesium, phosphorus, zinc, potassium, and iron, while red and brown rice are notably high in selenium, copper, and manganese. These rice types are also abundant in vitamins, particularly vitamin E and B vitamins, such as thiamine, riboflavin, niacin, and B6. Our literature survey indicated that black rice contains higher levels of γ-oryzanols, tocols, and folate compared to other rice types, while brown rice provides more vitamin B6 than black rice.

Our literature survey highlights that the analytical techniques such as GC-MS, LC-MS, and nuclear magnetic resonance are expanding the list of identified compounds in colored rice. This progress is revealing novel bioactive compounds with potential health benefits, especially in black rice, which has been the most extensively studied variety in terms of its nutritional and metabolomic profiles, bioactivities, and breeding.

**Figure 2:**
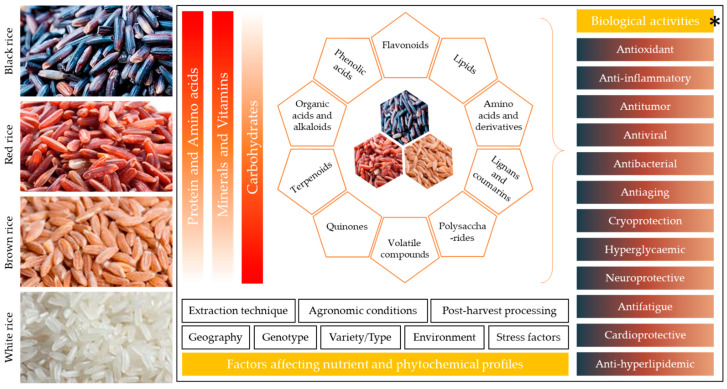
Summary figure showing trends of nutrients, major bioactive compounds, and bioactivities of different types of colored rice. The bars representing protein, amino acid, minerals, vitamins, and carbohydrates show red (maximum) and white (minimum) contents. Several factors can influence the nutrient and phytochemical profiles of the colored rice. The right panel shows biological activities associated with colored rice. The three colors in the biological activity bars indicate that black, red, and brown rice exhibit these biological activities in decreasing order. * Represents that the biological activities could depend on the metabolite type and its content in each type of rice being tested.

Health-promoting activities of colored rice include a range of biological effects that contribute to overall well-being. Black and red rice have demonstrated antioxidant, antimicrobial, anti-inflammatory, hypoglycemic, anti-aging, and neuroprotective properties (Figure 2). These benefits are concentration- and dose-dependent, highlighting the importance of proper intake levels for optimal health outcomes. Clinical studies, although limited in number, have shown promising results, suggesting that the regular consumption of colored rice may offer various therapeutic effects (Table 4). However, more long-term in vivo research is needed to fully understand the extent of these health benefits.

In conclusion, the rich and diverse nutritional profile of colored rice provides numerous health benefits and presents exciting opportunities for further research in breeding and functional food development. The unique combination of bioactive compounds, proteins, lipids, minerals, and vitamins in black, brown, and red rice not only makes them a valuable part of a balanced diet but also emphasizes their potential for future development and application in the food industry, particularly in areas related to functional foods and health-promoting products.

## 6. Future Research

Future research on colored rice should focus on several directions including breeding for higher bioactive compounds, balancing nutrition and yield, and developing varieties with specific color and nutritional traits. This literature survey showed that the anthocyanin biosynthesis pathway in colored rice is well understood, such that the major genes contributing towards pigment biosynthesis are known through transcriptomic studies. Moreover, the inheritance pattern of anthocyanin pigmentation is well understood [14]. These genes, together with their transcription factors and regulatory genes, are prime targets for the expanding the color range of rice grains to expand the consumer base [270]. In terms of nutrition, the main challenge is to balance nutrition and yield through artificial selection, mutation breeding [188], multi-omics, genomics-assisted breeding, marker-assisted selection, and genome editing [271,272]. While most research has focused on elucidating the flavonoids and anthocyanin pathways, other key metabolic pathways are the least explored; for example, information on the differential biosynthesis of phenolic compounds that are absent in black rice but present in red rice, e.g., benzamide and p-hydroxyphenyl acetic acid, and those that are exclusively present in black rice, e.g., chlorogenic acid methyl ester, is not available. Future studies employing co-joint transcriptome and metabolome approaches may allow researchers to associate key pathways and genes with differences in phenolic composition [106]. Such knowledge would be useful for improving the phenolic content/composition of colored rice varieties to make them better functional foods. Efforts should be made to develop colored rice varieties with lower carbohydrate contents and higher contents of the key health-beneficial compounds, and relatively higher lipid and protein contents. Most breeding efforts to understand the genetics of protein and carbohydrate content and composition focus on white rice, with limited or no studies on colored rice. For high-lipid-content rice, only a few studies on white rice have been published. This scenario highlights the future opportunities for using the knowledge accumulated in white rice, in terms of associated genes and quantitative trait loci, to improve colored rice. Moreover, the knowledge of the volatilome profile should be considered during breeding, as consumer preferences may differ for pigment rice with or without an aroma. While some metabolite classes have been studied in detail, others, such as alkaloids, lipids, and organic acids, have been less studied. For example, although in lesser quantities, the lipids in colored rice gains belong to diverse sub-classes and call for the application of other genomic and transcriptomic approaches, as well as GWAS, for delineating the respective pathways and loci involved. Such efforts may lead towards breeding colored rice cultivars with the required content and composition of lipids. Nevertheless, developments in analytical chemistry are extending the list of detected compounds, but their differential biosynthesis remains less explored. In terms of detection and quantification, it is important to emphasize that the comparative profiling using the same technique should be a way forward. In particular, it is important to follow the same set of experimental conditions, i.e., agronomy, light, temperature, humidity, and others, in order to critically compare the group of compounds present in each type. These aspects have been less considered in the comparative studies on global metabolomics. This would also help in improving our understanding of the use of several classes of compounds for classifying colored rice. However, care must be taken in this regard since agronomic conditions and other factors may result in different metabolome profiles, such that derivatives of the same metabolite/compound could be biosynthesized under different conditions [138].

With regard to pharmacological and clinical applications, although much research has been conducted, several avenues for future exploration are suggested. Future research should focus on the health-beneficial activities such as antibacterial, antiviral, neuroprotective, and other properties in relation to specific compounds. In particular, the antiviral effects of the compounds present in colored rice are less explored, indicating an opportunity for researchers. Notably, among the secondary metabolites, flavonoids, anthocyanins (e.g., cyanidin-3-O-glucoside), tocopherols, and tocotrienols have been extensively studied and linked with a range of biological activities [92,221]. With the increasing list of compounds identified, it is essential to test and correlate individual compounds with their respective bioactivities and to elucidate their key mechanisms of action. Moreover, when analyzing the health effects of multiple compounds at the same time, the interpretation of such capacity assays should be undertaken by keeping in mind the complex pathway interactions [164]. Apart from the beneficial pharmacological activities, it is essential to not neglect the toxicities of different compound classes, e.g., alkaloids [143]. In particular, the detection of >100 alkaloids [106] provides a tentative list of compounds for future research on determining the beneficial or toxic effects on human health. This would help in the development of recommendations on the consumption of each type of colored rice for disease or health conditions. However, key considerations for recommendations should include geography, disease/malnutrition, type of colored rice available in the market, gender, age, ethnicity, dietary pattern of consumer/patient, daily intake, and duration. Consumers should be well informed about the standardized cooking method for the recommended colored rice, as research has shown that some methods, such as steam cooking, offer least nutritional loss. This underlines the need for standardized cooking methods that specify the degree of cooking, temperature range, and processing time. Research on the digestibility and changes during digestion is scarce. In particular, future research should explore the digestibility of starch under the influence of different lipid profiles, the hydrolysis of starch [126], and how different bioactive compounds are metabolized to their derivatives or other compounds.

Almost all of the literature surveyed regarding the health-promoting activities of bioactive compounds in colored rice used methods involving in vitro assays. There is a limited quantity of clinical trial-based data on the use of pigmented rice. However, to maximize the benefits of the nutritional and bioactive profiles of the pigmented rice and to implement the knowledge in clinical practice, future studies need to focus on prolonged in vivo studies and clinical trials. A key observation in the surveyed literature is that the main focus of individual studies is on only one type of pigmented rice, so conclusions about the comparative clinical significance of different colored rice varieties cannot be drawn. Moreover, each study uses a different number of cohorts and different doses, concentrations, and forms of pigmented rice (cooked, extract, or boiled). Therefore, future studies must focus on long-term clinical trials on the use of pigmented rice, including different types of colored rice at similar doses. Moreover, such studies need to optimize and adapt standardized methods for measuring the biological activities. In selecting the most appropriate pigmented rice varieties, the effect of the genotype, environment, and their interaction should not be ignored. Such detailed long-term clinical trials would reveal useful data for applications in clinical practice.

From the point of view of industrial use, it should be noted that raw rice usually has a higher level of the bioactive compounds and therefore should be considered carefully to avail higher health benefits [186]. In terms of health benefits, efforts should be made to develop pharmaceutical products, extracts, nanoparticles [199], gels, sourdough [207], skin care products, food supplements, and concentrated isolates of bacteria [200].

## Data Availability

The original contributions presented in this study are included in the article. Further inquiries can be directed to the corresponding author.
